# Influence of DPYD*9A, DPYD*6 and *GSTP1 ile105val* Genetic Polymorphisms on Capecitabine and Oxaliplatin (CAPOX) Associated Toxicities in Colorectal Cancer (CRC) Patients

**DOI:** 10.31557/APJCP.2019.20.10.3093

**Published:** 2019

**Authors:** Ashok Varma K, M Jayanthi, Biswajit Dubashi, D G Shewade

**Affiliations:** 1 *Department of Pharmacology, *; 2 *Department of Medical Oncology, JIPMER, Puducherry, India. *

**Keywords:** CAPOX, Toxicities, Predictive markers, DPYD*9A, DPYD*6, GSTP1 ile105va, Colon cancer

## Abstract

**Aim:**

CAPOX treatment in CRC patients was reported to cause several dose-limiting toxicities, and are found responsible for treatment interruption or even discontinuation. Therefore there is a critical need for identifying the predictive biomarkers for such toxicities to prevent them. The aim of our present study is to find the influence of DPYD*9A, DPYD*6 and *GSTP1 ile105val *gene polymorphisms on CAPOX treatment-associated toxicities in south Indian patients with CRC.

**Patients and Methods:**

We have recruited 145 newly diagnosed and treatment naive CRC patients in the study. Each Patient received a standard treatment schedule of oxaliplatin 130 mg/m2 infusion over 2 hours on day 1 and oral capecitabine 1000mg/m^2^ in divided doses twice daily for the next 14 days of a 21-day cycle. 5 ml of the venous blood was collected from each patient and genomic DNA extraction and genotyping. The genotyping analysis of the selected genetic polymorphisms was carried out by real-time PCR using TaqMan SNP genotyping assays obtained from applied biosystems.

**Results:**

The major dose-limiting toxicities observed with CAPOX treatment were thrombocytopenia, HFS and PN. DPYD*9A carries were found to be at higher risk for HFS, diarrhoea and thrombocytopenia when compared to patients with wild allele. No significant association was found between DPYD*6, *GSTP1 ile105val *polymorphisms and CAPOX related toxicities except for thrombocytopenia.

**Conclusion:**

A significant association was observed between DPYD*9A polymorphism and CAPOX induced dose-limiting toxicities strengthening its role as a predictive biomarker.

## Introduction

5-fluorouracil and its oral prodrug capecitabine are the widely used anticancer agents and indeed their combination regimens with other anticancer drugs are the backbone in the treatment of various cancers like colon, rectum, breast, stomach, oesophagal and pancreatic cancer etc (Malet-Martino and Martino, 2002). Capecitabine and oxaliplatin combination regimen (CAPOX) is the standard chemotherapeutic care for the treatment of colorectal cancer (CRC) in the last decade. CAPOX treatment was proven to be equivalent or non-inferior to standard regimens like FOLFOX and FOLFRI in the treatment of advanced and metastatic CRC (Pectasides et al., 2015; Guo et al., 2016; Sobrero et al., 2018) and preferred over its counterparts due to convenience in administration and easy management. (Wehler et al., 2012) However, CAPOX treatment was associated with several dose-limiting haematological and non-haematological toxicities (Mullally et al., 2018; Baird et al., 2011) and patients show high inter-individual variation to its toxicity profile. (Haller et al., 2008) These toxicities may limit treatment effectiveness as they impose treatment interruption or even discontinuation and often require hospitalization which in turn increases health care costs. Therefore there is a critical need for identifying the predictive biomarkers for CAPOX related toxicities.

Dihydropyrimidine dehydrogenase (*DPYD*) is a rate-limiting enzyme and found to metabolize 80% of the administered capecitabine. (Caudle et al., 2013) *DPYD* gene was found to be genetically polymorphic and its deficiency was reported to cause severe toxicities with capecitabine treatment. The US food and drug administration (FDA) and European medical agency (EMA) approved drug label of capecitabine warns for the unexpected, severe toxicities like stomatitis, hand-foot syndrome (HFS), diarrhoea, mucosal inflammation, neutropenia in the deficiency of *DPYD* enzyme and states that no dose has been proven safe in patients with complete absence of *DPYD* enzyme. (Xeloda-Epar-Product-Information.; XELODA (Capecitabine) Tablets, for Oral Use,”) DPYD*2A polymorphism (1905+1 G>A splice donor variant) is a classic genetic variant and individuals who express this polymorphism were reported not to metabolize capecitabine at a normal rate and were found to be at a higher risk of developing severe life-threatening toxicities. (Toffoli et al., 2015; Deenen et al., 2016) However, the major limitation of DPYD*2A of being used as a predictive marker for toxicity is its lower minor allele frequency varying from 0.1 % to 1% in different ethnic groups. (Henricks et al., 2017) A recent study states that only a 50 % patients with DPYD*2A carriers actually develop toxicity with 5-FU treatment and reported that novel *DPYD* variants, DPYD*9 and DPYD*6 carry 2 fold higher risk for toxicities with 5 –FU treatment when compared to DPYD*2A polymorphism alone (Gentile et al., 2016). This emphasizes the search for novel genetic toxicity predictive markers for capecitabine treatment.

DPYD*9A (rs id 1801265) is a novel missense single nucleotide variant (A>G) located on chromosome 1, at position 97883329. The A>G replacement induces an amino acid change of cysteine to arginine in the coding region and found to alter the DPYD enzyme activity. DPYD*6 (rs id 1801160) is another missense single nucleotide variant (C>T) located in the chromosome 1, at position 97305364. The C>T replacement induces amino acid change valine to isoleucine. Both these polymorphisms were reported to alter the catalytic activity of *DPYD* enzyme and linked with capecitabine associated toxicities. (Offer et al., 2014; Gross et al., 2003; Baskin et al., 2015)

Glutathione-S-transferase P1 (*GSTP1*) is a rate-limiting enzyme involved in detoxification of oxaliplatin. It mediates oxaliplatin-glutathione conjugation (GSH) reaction for the easy elimination of oxaliplatin from the body through kidneys. GSTP*1 Ile105val (rs1695, A>G) is a missense single-nucleotide on chromosome 11 at position 67585218 and was found to lower the expression of *GSTP1* enzyme. Oxaliplatin-related cumulative neuropathy and neutropenia were reported to be more frequent and severe in patients with heterozygous (AG) and homozygous (GG) genotype when compared to wild allele (AA) patients (Lecomte et al., 2006; Zhong et al., 2006) The aim of our study was to find the association between *DPYD* and *GSTP1* gene polymorphisms and toxicities with CAPOX treatment in south Indian patients with colorectal cancer.

## Materials and Methods

The study was approved by the JIPMER scientific advisory committee (JSAC Reg.No.JSAC 34/6/2016) and JIPMER ethics committee (JEC Reg.No: 25-5-2016). In a prospective cohort study, we recruited 145 newly diagnosed and treatment-naive CRC patients from January 2016 to December 2018. Patients with age ≥ 18 years of either gender, who were scheduled to receive CAPOX as their standard treatment care were included in the study. Previously treated, pregnant, lactating women and patients with abnormal liver function (serum transaminases ≥ 2 times the normal value) or renal function (creatinine >1.5 g/dl) parameters were excluded from the study. Patients received regimens other than CAPOX were excluded from the study. The demographic details and patients characteristics like age, sex, cancer stage, treatment setting, comorbidities, smoking and drinking habits were collected at baseline. Apart from the above data, baseline haematological values, renal and liver function parameters, starting dose of CAPOX, any dose reduction or treatment delay or drug discontinuation and treatment-related deaths were recorded during each follow-up. Informed consent was obtained from all the patients.

Each patient received a standard treatment schedule of oral capecitabine 1,000 mg/m^2^ in divided doses twice daily for 14 days and oxaliplatin 150 mg/m^2^ infusion over 2 hours on day 1 of a 21-day cycle. The median number of CAPOX cycles administered was 12. During each cycle, the treatment-related toxicities were noted and analyzed. The toxicities were divided into haematological and non-haematological toxicities. All the toxicities are graded for severity by using common terminology criteria for adverse events (CTCAE). (“Common Terminology Criteria for Adverse Events” 2017)

*DNA extraction and genotyping*

5 ml of the venous blood was collected from each patient and subjected to centrifugation for 5 min at 2,500 g for plasma separation. Plasma was discarded and the pellets containing red blood cells (RBC) with the buffy coat of white blood cells (WBC) were stored at −20°C until DNA extraction. Genomic DNA was extracted from the WBC by the phenol-chloroform method. (“Shared Protocol-Extracting-DNA-Using-Phenol-Chloroform.Pdf” n.d.) The extracted DNA was analyzed qualitatively and quantitatively using biophotometer plus (Eppendorf AG 22331, Hamburg, Germany). Each DNA sample was diluted to an optimal concentration of 50 ng/μL suitable for further downstream analysis and stored in aliquots at 4°C. The genotyping analysis for the selected single nucleotide polymorphism (DPYD*9A, DPYD*6 and Ile105val A>G was carried out by real-time PCR (7300 Applied Biosystems; Life Technologies Corporation, Carlsbad, CA, USA) using TaqMan SNP genotyping assays (rs id 1801265, rs id 1801160, rs 1695) purchased from applied biosystems. Version 1.4 of 7300 sequence detection software (SDS) was used for absolute quantification and allelic discrimination (Kodidela et al., 2015)

**Table 1:** Baseline Characteristics of the Patients

S.no		No of Patients		
	Characteristics	Colon	Rectum	Total (%)
1	Gender			
	a Male	62	28	90 (62)
	b. Female	40	15	55 (38)
2	Age in years – mean ± SD	50±13	49±12	--
3	Ethnicity			
	a. Tamilian	82	29	111 (76.5)
	b. Andhra	11	9	11 (14.4)
	c. North Indians	9	5	7 (9.6)
4	Performance status			
	a. 0-1	76	30	106 (73.1)
	b. 2	18	9	27 (19)
	c. 3	8	4	12 (8)
5	Tumour site			
	a. Right colon	49	--	49 (33.7)
	b. Left colon	53	--	53 (36.5)
	c. Rectum	--	43	43 (29.6)
6	Cancer stage			
	a. II	22	4	26 (18)
	b. III	38	19	57 (39.3)
	c. IV	42	20	62 (42.7)
7	Chemotherapy setting			
	a. Neoadjuvant	5	8	13 (9)
	b. Adjuvant	55	15	70 (48.2)
	c. Palliative	42	20	62 (42.7)
8	Habits			
	a. Smoking	15	7	22 (15.1)
	b. Alcoholic	7	5	12 (8.2)
	c. Smoking+ Alcoholic	10	8	18 (12.4)
9	Comorbidities			
	a. Diabetes	10	8	18 (12.4)
	b. Hypertension	12	7	21 (14.4)
	c. Thyroid disorders	2	0	2 (1.3)

**Table 2:** Observed Genotype Frequency and Toxicity Frequency in CRC Patients (N=145)

			Toxicity frequency across genotypes –N (%)						
S.no	Gene & genotype	Freq	Anemia	TP	NP	Diarrhoea	Vomiting	HFS	PN
			64 (44%)	50 (35%)	31 (21%)	33(22%)	58 (40%)	63(43%)	46 (32%)
1	DPYD*9A								
	AA	100	45	28	20	14	30	26	26
	AG	35	12	17	6	12	22	29	17
	GG	10	7	5	5	7	6	8	3
2	DPYD*6								
	CC	122	54	41	24	22	37	42	32
	CT	18	8	7	6	8	18	15	12
	TT	5	2	2	1	4	3	3	2
3	GSTP1 ile105val								
	AA	70	28	18	14	28	15	32	21
	AG	57	23	22	10	18	10	21	13
	GG	18	8	10	7	12	8	10	12

TP, thrombocytopenia; NP, neutropenia; HFS, hand foot syndrome; PN, peripheral neuropathy.

**Table 3:** No of Patients with Dose Reduction or Treatment Delay or Drug Discontinuation due to Toxicities (N=145)

S.no	Toxicity	No of patients with dose reduction (%)	No of patients with treatment delay (%)	No of patients with drug discontinuation (%)
1	Anaemia	6 (4.1)	3 (2)	0 (0)
2	TP	9 (6.2)	4 (2.7)	4 (2.7)
3	HFS	8 (5.5)	3 (2)	6 (4.1)
4	PN	6 (4.1)	5 (3.4)	3 (2)
5	Diarrhoea	5 (3.4)	3 (2)	0 (0)
6	Vomiting	1 (0.6)	3 (2)	0 (0)
7	Infusional reaction	0 (0)	0 (0)	2 (1.3)
	Total	35 (24%)	21 (14%)	15 (10.3%)

**Table 4:** Association between Haematological Toxicities and Genetic Polymorphisms (DPYD*9A, DPYD*6 and GSTP1 ile105val)

S.no	Model	Genotype	Freq		Observed toxicity across genotypes		
					Anemia	Thrombocytopenia	Neutropenia
1	DPYD*9A	AA	100		45	28	20
		AG	35		12	17	6
		GG	10		7	5	5
	Dominant model	AA (ref) AG+GG	100 35+10	P-value Odds (CI)	0.8 0.9 (0.4-1.8)	0.01* 2.4 (1.18-5.1)	0.5 1.24 (0.5-2.9)
2	DPYD*6	CC	122		54	41	24
		CT	18		8	7	6
		TT	5		2	2	1
	Dominant model	CC(ref) CT+TT	122 18+5	P-value Odds (CI)	0.8 1.9 (0.4-2.6)	0.6 1.2 (0.5-3.1)	0.2 1.7 (0.6-1.7)
3	GSTP1 ile105val	AA	70		28	18	14
		AG	57		23	22	10
		GG	18		8	10	7
	Dominant model	AA (ref) AG+GG	70 57+18	P-value Odds (CI)	0.8 0.9 (0.4-1.8)	0.04 2 (1.1-4.1)	0.6 1.2 (0.5-2.6)

ref, Reference; *, Significant; The frequency of wild type allele (high frequency allele) was taken as reference for calculating the odds ratio in dominant model of genotyping analysis.

**Table 5:** Association between Non-Haematological Toxicities and Genetic Variants (DPYD*9A, DPYD*6 and GSTP1 ile105val)

S.no	Model	Genotype	Freq		Observed toxicity across genotypes			
					Vomiting	Diarrhoea	HFS	PN
1	DPYD*9A	AA	100		35	18	37	32
		AG	35		17	8	18	10
		GG	10		6	7	8	4
	Dominant model	AA (ref) AG+GG	100 35+10	P value Odds (CI)	0.06 1 (0.9-4)	0.04 * 2.7 (1.8-4)	0.02* 2.3 (1.8-4(	0.9 09 (0.4-2)
2	DPYD*6	CC	122		49	25	54	38
		CT	18		9	6	7	5
		TT	5		0	2	1	3
	Dominant model	CC (ref) CT+TT	122 18+5	P value Odds (CI)	0.9 1 (0.4-2)	0.1 1.8 (0.7-2)	0.3 0.6 (0.2-1)	0.7 1.1 (0.4-2)
3	GSTP1 ile105val	AA	70		28	15	32	21
		AG	57		18	10	21	13
		GG	18		12	8	10	12
	Dominant model	AA (ref) AG+GG	70 57+18	P value Odds (CI)	0.8 1 (0.4-2)	0.9 1 (0.4-2)	0.2 1 (0.3-1)	0.7 1.2 (0.5-2)

ref, Reference; *, Significant; The frequency of wild type allele (high frequency allele) was taken as reference for calculating the odds ratio in dominant model of genotyping analysis.

**Table 6:** Multinominal Regression Analysis between Covariates and Hematological Toxicities

S.no	Co-variate	Anemia	Thrombocytopenia	Neutropenia
		P value - Odds (CI)	P value-Odds (CI)	P value-Odds (CI)
1	Age	0.5- 1 (0.4-2)	0.01 – 2.5 (0.4-5)	0.08 – 2.3 (0.8-6)
	a. <50	Ref	Ref	Ref
	b. >50			
2	Sex			
	a. Male	0.9 –1 (0.5-2.2)	0.9 – 1 (0.4-2.3)	0.1 – 0.8 (0.1-2)
	b. Female	Ref	Ref	Ref
3	Ethnicity			
	a. Tamilian	0.4 - 0.6 (0.1-2.1)	0.5 – 1.4 (0.4-5)	0.7- 1.2 (0.2-6)
	b. Andhra	0.5 - 0.6 (0.1-2.4)	0.3 - 2 (0.4-9)	0.5 – 0.8 (0.1-3.5)
	c. North Indians	Ref	Ref	Ref
4	Performance			
	a. 0-1	0.07 -1.7 (0.1-3)	0.2 – 1.2 (0.4-1.3)	0.6 – 0.9 (0.1-4)
	b. 2	0.1 - 0.9 (0.3-1.8)	0.1 - 1 (0.6-1.7)	0.1 – 1 (0.6-3)
	c. 3	Ref	Ref	Ref
5	Setting			
	a. Adjuvant	0.5 – 1.4 (0.3-5)	0.9 – 1 (0.2-4)	0.2 – 1 (0.5-1.8)
	b. Palliative	Ref	Ref	Ref
6	Cancer			
	a. Colon	0.2 – 1.5 (0.7-3)	0.4 – 1.3 (0.6-2)	0.5 – 1.3 (0.4-3)
	b. Rectum	Ref	Ref	Ref
7	Cancer stage			
	a. II	0.3 – 0.5 (0.1-1.7)	0.6 – 1 (0.2-2)	0.8 – 1 (0.2 -4)
	b. III	0.9 – 0.9 (0.2-4)	0.9 – 1 (0.2-4)	0.2 – 1.5 (0.4- 5)
	c. IV	Ref	Ref	Ref

Ref, Reference; *, Significant

**Table 7:** Multinominal Regression Analysis between Covariates and Non-Hematological Toxicities

S.no	Co-variate	HFS	PN	Diarrhoea	Vomiting
		P value-Odds (CI)	P value-odds (CI)	P value-odds (CI)	P value-odds (CI)
1	Age				
	a. <50	0.851 -1.7 (0.5-2)	0.3 - 1.3 (0.6-2.9)	0.6 – 1.2 (0.5-2.7)	0.7 – 1.1 (0.5-2.3)
	b. >50	Ref	Ref	Ref	Ref
2	Sex				
	a. Male	0.615 -1.2 (0.5-2)	0.6 – 0.8 (0.3-1.8)	0.3 – 1.4 (0.6-3.5)	0.7 – 1.1 (0.5-2.4)
	b. Female	Ref	Ref	Ref	Ref
3	Ethnicity				
	a. Tamilian	0.26 - 1.4 (0.6-2)	0.8 – 1.1 (0.3-3.9)	0.85 - 0.8 (0.2-3)	0.9 – 1 (0.2-3.4)
	b. Andhra	0.19 - 1.6 (0.6-2)	0.4 – 1.8 (0.3-8.6)	0.13 -0.29 (0.4-1)	0.4 – 0.5 (0.1-2.4)
	c. North Indians	Ref	Ref	Ref	Ref
4	Performance				
	a. 0-1	0.09 - 0.5 (0.2-1)	0.4 – 0.5 (0.7-3)	0.4 - 0.9 (0.7-3)	0.6 – 1.5 (0.2-3)
	b. 2	0.2 - 1 (0.3-2)	0.9 – 1 (0.14-6)	0.8 - 0.7 (0.1-4)	0.1 - 1.2 (0.2-1.2)
	c. 3	Ref	Ref	Ref	Ref
5	Setting				
	a. Adjuvant	0.1 - 1.1 (0.7-1)	0.8 – 1 (0.19-3)	0.3 – 1.3 (0.4-2)	0.4 – 1.4 (0.7-3.4)
	b. Palliative	Ref	Ref	Ref	Ref
6	Cancer				
	a. Colon	0.8 - 1.3 (0.3-4)	0.2 – 1.5 (0.7-3)	0.9 – 1 (0.4-2)	0.16 – 0.5 (0.2-1)
	b. Rectum	Ref	Ref	Ref	Ref
7	Cancer stage				
	a. II	0.8 - 0.9 (0.2-2)	0.7 – 0.8 (0.2-2.7)	0.07 – 0.6 (0.1-1)	0.2 – 0.9 (0.6-1.2)
	b. III	0.7 - 1.2 (0.3-5)	0.9 – 1 (0.2-5.1)	0.9 – 1 (0.1-5)	0.7 – 1.6 (0.8-2.4)
	c. IV	Ref	Ref	Ref	Ref

Ref, Reference; *, significant; HFS, hand foot syndrome; PN, peripheral neuropathy

**Diagram1:**
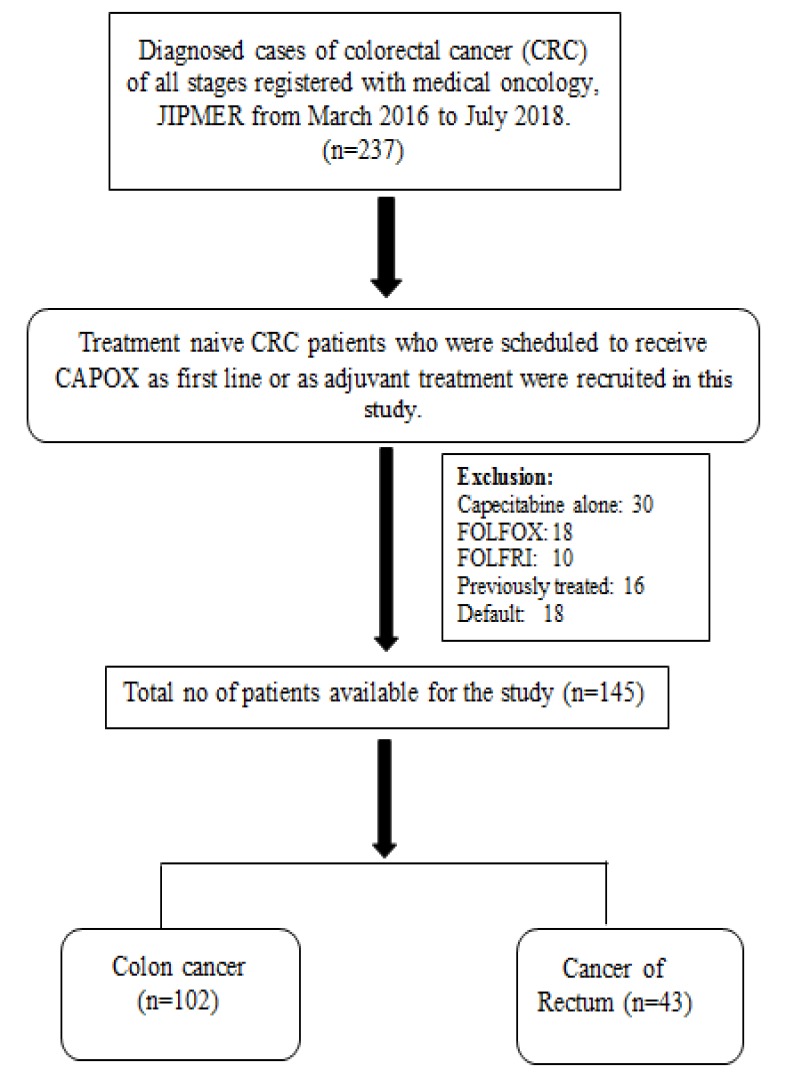
Flow- Diagram

*Toxicity grading and Statistics*

All the toxicities are graded according to common terminology criteria of adverse effects version 3.0 (CTCAE). Demographic parameters were expressed as mean ± Standard deviation. Adverse effects are represented in percentages and analyzed descriptively. Genotyping frequencies of the selected polymorphisms were analyzed for hardy Weinberg equilibrium. The association between genetic variants and CAPOX related toxicities were analyzed by chi-square association test using SPSS version 19.0 (IBM-SPSS). Multinomial regression analysis is done to find the influence of confounding factors on toxicities. A p-value of less than 0.05 was considered to be statically significant.

## Results

The number of male patients was 90 with a mean age of 50 ± 13 and female patients were 55 with a mean age of 49 ± 12. The number of patients diagnosed with colon cancer were 102 and with the rectum cancer were 43. Most of the patients received CAPOX as an adjuvant treatment (48.2%) or palliative care (42%). The median number of CAPOX cycles administered were 12 and the median follow up time was 18 months. Other baseline characteristics of the patients included in the study are tabulated in Table 1. 

The genotyping frequencies of DPYD*9, DPYD*6 and *GSTP1 ile105val *polymorphisms were in Hardy Weinberg Equilibrium. The most frequently observed haematological toxicities were anaemia, thrombocytopenia (TP) and neutropenia (NP), whereas vomiting, HFS and PN are the frequently observed non-haematological toxicities. The major dose-limiting toxicities were thrombocytopenia, HFS and PN. A total of 24% of the patients needed dose reduction, 14% of the patients needed treatment delay and 10% of the needed drug discontinuation due to toxicities. (Table 2 and 3).

In a dominant model of genotyping analysis, we found a significant association between DPYD*9A polymorphism and capecitabine related toxicities strengthening its role as a predictive biomarker. Patients with DPYD*9A polymorphism had a 2.4 (CI: 1.18-5.1) times higher risk for thrombocytopenia, 2.7 (CI: 1.8-4) times higher risk for diarrhoea and 2.3 (CI: 1.8-4.7) times higher risk for HFS when compared to wild type patients. No significant association was found between DPYD*6 polymorphism and capecitabine related toxicities. Similarly, we did not find any significant association between *GSTP1 ile105val *polymorphism and oxaliplatin-related toxicities except for thrombocytopenia. Patients with *GSTP1 ile105val *polymorphism had 2 (CI: 1.1-4.1) times higher risk for thrombocytopenia when compared to wild type (Table 4 and 5).

We also performed a multinomial logistic regression analysis to find the influence of covariates such as age, sex, ethnicity, patient’s performance, cancer type, treatment setting and cancer stage on CAPOX related toxicities. We dint find any significant association between the covariates and toxicities except for age and thrombocytopenia. Patients with age group <50 years had shown a significant association with thrombocytopenia when compared with an age group >50 (Table 6 and 7). 

## Discussion

The adverse drug effects (ADE) associated with cancer chemotherapy are a real concern for the patients and clinicians as they cause treatment interruption or even discontinuation. The current strategies of toxicity management with anticancer drugs either follow a holistic approach and nor addresses the long term complications. Looking for the inter-individual genetic makeup of an individual is a novel approach for predicting the toxicities associated with anticancer drugs. Studying genetic alterations, mainly the genes coding for the drug-metabolizing enzymes can serve as an important tool in identifying predictive biomarkers for drug-related toxicities. The prior screening and adjusting the dose in such patients can decrease the ADE rate.

In the present study, we looked for the adverse effects related to CAPOX treatment and their association with *DPYD* and *GSTP1* gene polymorphisms. Thrombocytopenia, HFS and PN were the major dose-limiting toxicities observed with CAPOX treatment. HFS is a characteristic side effect with capecitabine with the symptoms ranging from mild blackish skin discolouration to severe skin changes like peeling, blisters, bleeding and pain mainly in the palm of the hands and sole of the feet. (Lassere and Hoff, 2004) PN is dose-limiting toxicity associated with oxaliplatin and occurs due to drug accumulation either in the sensory or motor neurons. The involvement of sensory neurons often results in disturbing sensations like numbness, burning and shooting pain in the affected areas. Motor involvement often causes muscle weakness and paralysis. (Saif and Reardon, 2005) 

The implementation consortium guidelines (CPIC) of 2017 on *DPYD* genotyping states that DPYD*9A polymorphism reduces the enzyme activity however it doesn’t affect in a clinically relevant manner and limited its utility as a predictive toxicity biomarker. (Caudle et al., 2013) In the present study, we found a significant association between DPYD*9A polymorphism and capecitabine related toxicities strengthening its role as a predictive biomarker. The dominant model of genotyping analysis (AA vs AG+GG) has shown that heterozygous (AG) and homozygous (GG) carriers have a higher risk for HFS, diarrhoea and thrombocytopenia when compared to wild type (AA) carriers. Supporting to our study findings a recent study by Kushman et al. reported a significant association between DPYD*9A polymorphism and fluoropyrimidine induced toxicities in patients with gastrointestinal malignancies and recommend the oncologists to consider regular DPYD*9A screening along with other potential *DPYD* gene polymorphisms like DPYD*2A, 13 A and 9B (Khushman et al., 2018).

The available data on DPYD*6 polymorphism as a predictive biomarker is limited and conflicting. The DPYD implementation consortium guidelines (CPIC) of 2017 states that DPYD*6 presence may not always result in toxicity and its association with toxicities was not consistently replicated (Caudle et al., 2013) However, a recent study by Del Re et al., (2019) reported a 29% reduction in the DPYD enzyme activity in presence DPYD*6 polymorphism when compared to wild type and found a significant association with capecitabine related adverse effects. They also suggest for preemptive analysis for DPYD*6 polymorphism and 20% dose reduction in the homozygous variants and close monitoring of heterozygous variants. A study by Gentile et al., (2016) also reported that DPYD*6 and DPYD*9A are in strong haplotype association (hap 7) and their presence carries 2 fold higher risk of 5-FU toxicity compared to DPYD*2A polymorphism alone in the Italian population. However, in the present study, we observed no significant association between DPYD*6 polymorphism and capecitabine related toxicities. The lack of association may be due to observed low frequency of DPYD*6 hetero and homozygous mutants in our study cohort.

*GSTP1 ile105val *is one of the widely studied variants and has been highly linked for causing oxaliplatin-induced PN. Several independent studies reported a significant association between *GSTP1 ile105val *polymorphism and oxaliplatin-related PN (Lecomte et al., 2006; Chen et al., 2010; Kumamoto et al., 2013). However, a meta-analysis which is based on twelve prospective trials and two retrospective trials reported for no significant association between *GSTP1 ile105val *polymorphism and oxaliplatin-induced cumulative PN in allele dominant model and recessive model of analysis (Peng et al., 2013). Our study results are consistent with the meta-analysis data. We didn’t find any significant association between *GSTP1* le105val polymorphism and oxaliplatin-induced PN in the dominant model of analysis. 

In conclusion Thrombocytopenia, HFS and PN were the major dose-limiting toxicities with CAPOX regimen. A significant association was observed between DPYD*9A polymorphism and CAPOX induced toxicities like HFS, diarrhoea and thrombocytopenia strengthening its role as a predictive biomarker. No significant association was found between DPYD*6, *GSTP1 ile105val *polymorphisms and CAPOX induced toxicities. 
